# Associations Between Solitary Masturbation Parameters and Sexual Arousal in Partnered Sexual Activity

**DOI:** 10.3390/bs16071176

**Published:** 2026-07-13

**Authors:** Gracia M. Sánchez-Pérez, Juan Carlos Sierra

**Affiliations:** Mind, Brain and Behavior Research Center (CIMCYC), University of Granada, 18011 Granada, Spain; graciasp@ugr.es

**Keywords:** masturbation parameters, sexual arousal, partnered sexual activity, sexual functioning, complementary and compensatory models

## Abstract

Given the limited empirical evidence regarding the relationship between masturbation and sexual functioning, this study aimed to examine the association between multiple masturbation parameters—age of first masturbation, negative attitudes toward masturbation, solitary sexual desire, current frequency, and the intensity of the subjective orgasm experience—and self-reported sexual arousal within partnered sexual activity. Drawing on complementary and compensatory models, gender differences in these associations were also explored. A total of 1028 Spanish cisgender adults (469 men and 559 women; aged 18–70 years) who had engaged in heterosexual sexual activity and reported prior masturbation experience completed standardized measures assessing masturbation-related variables and sexual functioning. Separate multiple regression analyses were conducted for men and women, including self-reported overall sexual arousal and self-reported genital responses (penile erection and vaginal lubrication) as outcome variables. In men, the regression models explained a small proportion of the variance. Solitary sexual desire emerged as a positive predictor of self-reported sexual arousal, whereas no masturbation parameters were associated with self-reported penile erection, which was negatively predicted by age. In women, the models showed greater explanatory power. Self-reported sexual arousal was positively predicted by solitary sexual desire and the sensory dimension of the subjective orgasm experience. Self-reported vaginal lubrication was positively associated with these same variables and negatively associated with negative attitudes toward masturbation. Overall, the findings suggest gender-differentiated patterns in the relationship between masturbation and sexual arousal. In men, results are partially consistent with a compensatory framework, whereas in women they support a complementary model, highlighting the role of subjective and attitudinal factors in sexual functioning. These results contribute to a more nuanced understanding of masturbation and its relevance to sexual health, emphasizing the importance of considering multiple dimensions of sexual experience.

## 1. Introduction

Masturbation has historically been the subject of considerable debate, with cultural and historical stigmas contributing to the spread of misinformation and shaping social attitudes in the absence of robust scientific evidence (Eskandar, 2026). Although this sexual practice has often been regarded as less relevant than sexual relationships (Prytz, 2024), there is a growing need to consider a range of influencing factors to better understand this behavior and contribute to its destigmatization (Henriques et al., 2025).

Although masturbation has been recognized as a means of achieving sexual health (Burri & Carvalheira, 2019), research on its association with sexual functioning remains limited (Rowland et al., 2020; Soares et al., 2024). Nevertheless, existing evidence suggests that the relationship between masturbation and partnered sexual activity is complex, underscoring the need for further investigation into this topic (Cervilla et al., 2024a).

The relationship between solitary masturbation and partnered sexual activity should be interpreted within the frameworks of complementary and compensatory models. The compensatory model suggests that the frequency of masturbation increases in response to dissatisfaction with sexual relationships (Das et al., 2009; Regnerus et al., 2017). In contrast, the complementary model posits that masturbation and partnered sexual activity are not mutually exclusive, but may coexist and even mutually reinforce one another (Gerressu et al., 2008). Empirical evidence indicates a gender-differentiated pattern in the application of these models: the compensatory pattern appears to be more prevalent in male sexuality (Cervilla et al., 2025a; Fischer & Træen, 2022; Gerressu et al., 2008), whereas the complementary model has been more frequently associated with female sexuality (Gerressu et al., 2008; Regnerus et al., 2017).

Given the complexity of the relationship between solitary masturbation and partnered sexual activity, it is important to examine how different parameters of solitary masturbation are associated with various dimensions of sexual functioning within the context of sexual relationships, particularly sexual arousal. In the present study, both overall sexual arousal and specific genital response (i.e., erection in men and lubrication in women) are assessed using self-reported measures.

Research on masturbation has traditionally focused on the frequency of occurrence; however, in recent years, a broader set of parameters has been proposed to capture this behavior more comprehensively. These parameters include age of first masturbation, negative attitudes toward the behavior, solitary sexual desire, current masturbation frequency, and the intensity of the subjective orgasm experience (Cervilla & Sierra, 2022; Landaluce et al., 2026; Sánchez-Pérez et al., 2026). Considering these variables allows for a more holistic understanding of masturbation’s role in sexual functioning.

An earlier age of first masturbation may be associated with more favorable sexual arousal outcomes in adulthood (Carvalheira & Leal, 2013), similarly to how an earlier onset of sexual interest has been linked to a greater propensity for sexual arousal (Walton & Bhullar, 2018). Evidence also indicates that negative attitudes toward masturbation are related to lower levels of subjective sexual arousal (Mosher & Abramson, 1977), as well as greater difficulties in achieving erection (Cervilla et al., 2021; Sierra et al., 2021) and vaginal lubrication (Cervilla et al., 2021). More broadly, such attitudes have been linked to poorer sexual functioning (Cervilla et al., 2021; Soares et al., 2024).

Solitary sexual desire has consistently been positively associated with sexual arousal (Santos-Iglesias et al., 2013), erection (Sierra et al., 2021) and with the propensity for sexual arousal (Peixoto et al., 2018; Vallejo-Medina et al., 2020). In contrast, findings regarding masturbation frequency are heterogeneous. In men, higher frequency has been associated with both improved erectile response (Huang et al., 2022) and increased erectile difficulties (Niu et al., 2023). In women, higher frequency has been associated with greater ease in achieving sexual arousal (Carvalheira & Leal, 2013), although some studies report no association with sexual functioning (Soares et al., 2024). Additionally, relationship status may moderate these associations, with a negative relationship observed in partnered women and a positive relationship in single women (Huang et al., 2022). Similarly, higher masturbation frequency in women has been associated with fewer difficulties in achieving sexual arousal (Rowland et al., 2020). In both women and men, masturbation frequency has also been associated with a greater propensity for sexual arousal (Walton & Bhullar, 2018) and higher levels of subjective sexual arousal (Mosher & Abramson, 1977).

The subjective orgasm experience in the masturbation context has been conceptualized as a multidimensional construct, encompassing affective, sensory, intimacy, and rewards dimensions (Cervilla et al., 2024b). In men, the rewards dimension has been positively associated with the propensity for sexual arousal, whereas the intimacy dimension has been linked to higher ratings of sexual arousal. In women, the sensory dimension has shown a positive association with sexual arousal ratings (Cervilla et al., 2024b). Furthermore, in both men and women, greater orgasm intensity during masturbation has been associated with higher levels of sexual arousal and improved erectile or lubrication responses (Cervilla et al., 2025b).

To date, the association between masturbation parameters and sexual arousal, as well as genital responses such as penile erection and vaginal lubrication within sexual relationships, has not been comprehensively examined. Additionally, complementary and compensatory models have primarily been applied to the study of sexual satisfaction, with limited attention devoted to other outcomes, such as sexual arousal. In response to this gap, the present study seeks to expand current knowledge in this area. Specifically, and in line with the need to examine multiple factors in order to deepen understanding of masturbation and contribute to its destigmatization (Henriques et al., 2025), this study aims to analyze the relationship between different masturbation parameters (i.e., age of first masturbation, negative attitudes toward this behavior, solitary sexual desire, current masturbation frequency, and the intensity of the subjective orgasm experience) and self-reported sexual arousal in partnered sexual activity.

Based on the complementary and compensatory models, it is hypothesized that the association between masturbation and sexual arousal within partnered sexual activity will differ by gender. In men, a compensatory pattern is expected (Fischer & Træen, 2022; Gerressu et al., 2008), such that masturbation parameters may not be associated with enhanced sexual arousal. In contrast, in women, a complementary pattern is anticipated (Gerressu et al., 2008; Regnerus et al., 2017), whereby masturbation parameters are expected to be positively associated with sexual arousal.

## 2. Materials and Methods

### 2.1. Participants

A non-probabilistic sampling method was employed to recruit a total of 1028 Spanish adults (469 men and 559 women) aged 18 to 70 (*M* = 36.51; *SD* = 11.23). The mean age was 40.48 for men and 33.14 for women. In terms of educational attainment, 2.4% had completed primary education, 26.2% had completed secondary education, and 71.4% held a university degree. Additionally, 79.7% of the sample reported being in a romantic relationship.

The inclusion criteria were as follows: (a) being 18 years of age or older, (b) having engaged in heterosexual sexual activity within the past three months, and (c) having engaged in solitary masturbation at least once in their lifetime.

Table 1 shows the sexual history characteristics of the participants.

### 2.2. Procedure

Data collection was conducted between September 2025 and December 2025 using an online survey designed in LimeSurvey^®^ software (version 6.5.18+240723, Limesurvey GmbH, Hamburg, Germany). The survey was disseminated through social media and various digital platforms (e.g., X^®^, Instagram^®^, Facebook^®^, Telegram^®^, LinkedIn^®^), where digital posters were used to promote participation. Participants accessed the survey via a web link. Upon entry, respondents were required to complete a Completely Automated Public Turing test to tell Computers and Humans Apart (CAPTCHA), consisting of a randomly generated arithmetic operation designed to prevent automated responses.

A total of 6858 individuals accessed the survey. Of these, 4897 provided informed consent, and 2468 completed the survey in full. The dataset was subsequently screened according to the predefined inclusion criteria. Additionally, three attention-check items were included, and responses were reviewed to identify and exclude inconsistent or invalid data. Following this data-cleaning process, the final sample comprised 1028 participants.

The study was approved by the Ethics Committee on Human Research of University of Granada (Ref. 2984/CEIH/2022), ensuring compliance with ethical standards regarding participants’ rights, data protection, and confidentiality. No financial compensation was provided to participants.

### 2.3. Instruments

Sociodemographic and Sexual History Questionnaire. An ad hoc questionnaire was developed to collect information on sex assigned at birth, gender, age, nationality, educational level, type of sexual relationships (ranging from exclusively heterosexual to exclusively homosexual), current partner status, age of first masturbation, current masturbation frequency, and sexual relationships within the past three months.

The Spanish version of Negative Attitudes Toward Masturbation Inventory (Mosher, 2011) adapted by Cervilla et al. (2021) was used to assess negative attitudes toward masturbation. The instrument consists of 10 items (e.g., “I feel guilty about masturbating”) rated on a 5-point Likert-type scale ranging from 1 (*not at all true*) to 5 (*extremely true*), with higher scores indicating more negative attitudes. Previous research has reported excellent internal consistency (α = .95, and adequate validity evidence) (Cervilla et al., 2021). In the present study, McDonald’s omega coefficients were .89 for men and .90 for women.

Solitary sexual desire was assessed using the corresponding subscale of the Spanish version of the Sexual Desire Inventory (Spector et al., 1996), validated by Moyano et al. (2017). This subscale comprises four items (e.g., “During the last month, how often would you have liked to behave sexually by yourself, for example, masturbating, touching your genitals, etc.?”) with response options on varying Likert-type scales (e.g., 0 = *not at all* to 7 = *more than once a day*). Higher scores indicate greater solitary sexual desire. Previous studies have reported internal consistency coefficients above .90, and the adequate validity evidence (Moyano et al., 2017). In the present study, McDonald’s omega coefficients were .84 for men and .87 for women.

The Spanish version of the Orgasm Rating Scale (Arcos-Romero et al., 2018; Mah & Binik, 2011), validated for the masturbation context by Cervilla et al. (2022), was used to assess the subjective orgasm experience. This instrument included 25 adjectives grouped into four factors: Affective (i.e., feelings experienced during orgasm; e.g., “blissful”), Sensory (i.e., physiological sensations; e.g., “throbbing”), Intimacy (i.e., intimate aspects of orgasm; e.g., “loving”), and Rewards (i.e., rewarding effects; e.g., “soothing”). Items are rated on a 6-point Likert scale ranging from 0 (*does not describe it at all*) to 5 (*describes it perfectly*). Reported internal consistency ranges from .71 (Intimacy) to 0.95 (Sensory), with adequate validity evidence (Cervilla et al., 2022). In the present study, McDonald’s omega coefficients were .91 for men and .91 for women for the affective dimension, 0.95 (men) and 0.95 (women) for sensory dimension, .77 (men) and .74 (women) for intimacy, and .84 (men) and .81 (women) for rewards.

Sexual function was assessed using the Spanish version of the Arizona Sexual Experience Scale (McGahuey et al., 2000), validated by Sánchez-Fuentes et al. (2019). The scale included five items measuring sexual drive, arousal, penile erection/vaginal lubrication, ability to reach orgasm, and satisfaction from orgasm. Responses are rated on a 6-point Likert scale ranging from 1 (*hyperfunction*) to 6 (*hypofunction*). Scores were reverse-code so that higher scores indicate better sexual functioning. Previous research has reported internal consistency coefficients of .81 for men and .79 for women (Sánchez-Fuentes et al., 2019). Although was designed as a multidimensional scale, only the items assessing arousal (i.e., “How easily are you aroused?”) and penile erection/vaginal lubrication (i.e., “Can you easily get and keep an erection?” or “How easily does your vagina become moist or wet during sex?”) were included in the present analyses. In the Spanish validation study, these items showed high item-total correlations (*r* = .55–.83) and robust factor loadings (λ = .71–.78) in exploratory factor analyses (Sánchez-Fuentes et al., 2019), suggesting that they capture a substantial proportion of the variance in their respective constructs, even when analyzed independently.

### 2.4. Data Analysis

Missing data were handled using the missForest package (version 1.5; Stekhoven & Bühlmann, 2012) implemented in RStudio^®^ (version 2022.07.2+576; R Studio Team, 2020). The algorithm imputes missing values using random forest models fitted on observed data and iteratively updating the imputed values until the stopping criterion is reached; that is, when the difference between the newly imputed data matrix and the previous one increases for the first time. Although missForest relies on an ensemble of decision trees, it is designed to converge toward a single, robustly imputed data matrix (Stekhoven & Bühlmann, 2012).

To examine the associations between masturbation parameters and sexual arousal outcomes, separate multiple regression analyses were conducted for men and women, in line with the study’s theoretical framework proposing gender-specific patterns based on complementary and compensatory models. The dependent variables were overall sexual arousal and specific genital responses (i.e., penile erection in men and vaginal lubrication in women). Age was included as a covariate to control for its potential confounding effect. The following variables were entered as predictors in all models: age of first masturbation, negative attitudes toward masturbation, solitary sexual desire, current masturbation frequency, and the dimensions of the subjective orgasm experience. Statistical analyses were conducted using RStudio^®^.

## 3. Results

Separate multiple regression analyses were conducted to examine the association between masturbation parameters and self-reported sexual arousal outcomes in men.

The overall regression model predicting self-reported sexual arousal was statistically significant, *F*(9, 454) = 2.39, *p* = .012, although it explained a relatively small proportion of the variance (adjusted *R*^2^ = 0.026). As shown in Table 2, solitary sexual desire emerged as a significant positive predictor of self-reported sexual arousal (β = 0.20, *p* < .001), indicating that higher levels of solitary sexual desire were associated with increased sexual arousal in partnered sexual activity.

Regarding self-reported penile erection, the regression model was also statistically significant, F(9, 454) = 3.44, *p* < .001 (adjusted *R*^2^ = .045). Age was identified as a significant negative predictor (β = −.24, *p* < .001), suggesting that older age was associated with lower erectile response. No masturbation-related variables significantly predicted self-reported penile erection in the final model.

Separate multiple regression analyses were conducted to examine the association between masturbation parameters and self-reported sexual arousal outcomes in women.

The overall regression model predicting self-reported sexual arousal was statistically significant, *F*(9, 534) = 8.60, *p* < .001 (adjusted *R*^2^ = .112). As shown in Table 3, self-reported sexual arousal was positively predicted by solitary sexual desire (β = .24, *p* < .001) and the sensory dimension of the subjective orgasm experience (β = .12, *p* = .032). These findings indicate that higher levels of solitary sexual desire and more intense sensory orgasm experiences during masturbation were associated with greater sexual arousal in partnered sexual activity.

Regarding vaginal lubrication, the regression model was also statistically significant, *F*(9, 534) = 4.89, *p* < .001 (adjusted *R*^2^ = .061). Self-reported vaginal lubrication was positively predicted by solitary sexual desire (β = .16, *p* = .006) and the sensory dimension of the subjective orgasm experience (β = .11, *p* = .043) and negatively predicted by negative attitudes toward masturbation (β = −.09, *p* = .031). These findings suggest that higher levels of solitary sexual desire and stronger sensory orgasm experiences are associated with better genital response, whereas more negative attitudes toward masturbation are related to decreased self-reported vaginal lubrication.

## 4. Discussion

This study aimed to examine the relationship between multiple masturbation parameters (i.e., age of first masturbation, negative attitudes toward masturbation, solitary sexual desire, current frequency, and the intensity of the subjective orgasm experience) and self-reported sexual arousal in the context of partnered sexual activity, including both self-reported general sexual arousal and self-reported specific genital responses (i.e., penile erection and vaginal lubrication). In line with the complementary and compensatory models (Das et al., 2009; Regnerus et al., 2017), the findings reveal gender-differentiated patterns that appear differentially consistent with the expected frameworks. Specifically, the findings in women are more consistent with a complementary model, whereas the associations between masturbation parameters and partnered sexual arousal were limited and weak in men, suggesting specific nuances regarding the expected compensatory trend.

In men, self-reported sexual arousal showed association with solitary sexual desire, which partially contradicts the expected compensatory pattern, where no association or a negative relationship would be anticipated. However, the remaining masturbation parameters were not related to self-reported sexual arousal, and none were associated with self-reported penile erection, which appears more consistent with the expected compensatory framework. In contrast, in women, the results appear more consistent with the proposed complementary framework. Self-reported sexual arousal was positively associated with solitary sexual desire and the sensory dimension of the subjective orgasm experience, whereas self-reported vaginal lubrication was positively associated with these same variables and negatively associated with negative attitudes toward masturbation. Overall, these findings suggest that masturbation parameters play a more salient role in female sexual arousal and genital response.

Across both sexes, solitary sexual desire emerged as a significant correlate of self-reported sexual arousal. However, it was not associated with self-reported penile erection in men, whereas it was associated with self-reported vaginal lubrication in women. This discrepancy may be partly explained by differences in the nature of the dependent variables. As noted by Chivers et al. (2010), sexual arousal is a broader, more global construct, whereas penile erection represents a more specific and objectively identifiable physiological response. Although vaginal lubrication is also a physiological response, it is less directly observable and may be more closely linked to the subjective sexual experience. In this regard, men tend to evaluate their sexual arousal based on clearly identifiable physiological cues, such as erection, whereas women’s sexual responses may rely more on subjective and contextual factors (Baumeister, 2000; Chivers et al., 2010).

Furthermore, solitary sexual desire has consistently been associated with sexual arousal (Peixoto et al., 2018; Santos-Iglesias et al., 2013; Sierra et al., 2021; Vallejo-Medina et al., 2020), reinforcing its role as a central component in broader sexual functioning. The fact that solitary sexual desire was associated with self-reported sexual arousal in men also reflects a partial overlap between desire and arousal processes. Although this overlap has traditionally been more strongly documented in women (American Psychiatric Association, 2022; Brotto et al., 2009), the present findings suggest that it may also be relevant in men. Conceptually, solitary sexual desire is the parameter most closely aligned with self-reported sexual arousal, which may explain its stronger associative value compared to other masturbation-related variables. Overall, this pattern suggests that masturbation parameters are more strongly associated with global aspects of sexual functioning than with specific physiological responses, such as erection. A possible explanation for the absence or weakness of associations in men, particularly regarding self-reported penile erection, may be derived from clinical literature on sexual functioning. Some authors have noted that individuals may develop idiosyncratic masturbation styles, characterized by specific patterns of stimulation that are not easily replicated in partnered sexual contexts (Perelman, 2016). In such cases, discrepancies between solitary and partnered stimulation may limit the transfer of sexual arousal and may be related to physiological responsiveness during partnered sex. From a sexual conditioning perspective, repeated exposure to specific forms of stimulation may reinforce learned arousal patterns that do not readily generalize to different sexual contexts. Consistent with this interpretation, previous literature has suggested that frequent idiosyncratic masturbation, particularly when accompanied by fantasy patterns that differ substantially from those involved in partnered sex, may be associated with erectile difficulties (Porto, 2016).

In women, a greater number of masturbation parameters were associated with both self-reported sexual arousal and self-reported vaginal lubrication. This finding is consistent with previous research suggesting a stronger involvement of masturbation parameters in female sexuality (Cervilla & Sierra, 2022; Sánchez-Pérez et al., 2025), and is consistent with the complementary model, whereby masturbation and partnered sexual functioning appear to align and coexist positively (Gerressu et al., 2008; Regnerus et al., 2017). Additionally, this pattern may reflect the greater plasticity typically described in female sexuality (Baumeister, 2000, 2004), which could facilitate the integration of multiple experiential and attitudinal factors.

Beyond solitary sexual desire, negative attitudes toward masturbation and the sensory dimension of the subjective orgasm experience were also associated with female sexual functioning. Specifically, negative attitudes toward masturbation were associated with lower levels of vaginal lubrication, which is consistent with previous findings linking such attitudes to difficulties in female lubrication (Cervilla et al., 2021). However, these findings should be interpreted in light of the sample characteristics. Individuals who volunteer for sexuality research tend to differ systematically from those who do not, particularly regarding their comfort discussing sexual topics and their sexual attitudes (Arcos-Romero et al., 2025; Wiederman, 1999). Thus, the present sample may represent a subgroup with relatively more permissive or less variable attitudinal profiles, which could have influenced the observed associations. Therefore, caution is warranted when generalizing these findings to populations with greater diversity in sexual beliefs and norms. Conversely, the sensory dimension of the subjective orgasm experience in masturbation is positively associated with both self-reported sexual arousal and self-reported vaginal lubrication in partnered sexual activity, suggesting that greater attunement to bodily sensations during masturbation may be positively linked to sexual functioning in partnered contexts.

This finding aligns with research indicating that attention to bodily sensations plays a key role in facilitating sexual arousal, particularly in women (Silverstein et al., 2011). For example, Brotto et al. (2012) found that mindfulness-based interventions, which promote awareness of bodily sensations, were associated with increased sexual arousal. In this sense, the present results highlight the importance of moving beyond goal-oriented perspectives focused exclusively on enhancing arousal and instead considering how the capacity to connect with one’s own bodily sensations contributes to a more adaptive and satisfying sexual experience.

It is important to note that masturbation parameters represent only one subset of the factors potentially associated with different measures of sexual arousal. Thus, although the explanatory power of the models is modest, sexual functioning should not understood in isolation. Rather, it is a complex phenomenon in which biological, psychological, relational, and social dimensions are interdependent and intertwined (Basson, 2005; Brotto et al., 2016).

### Limitations

This study is not without limitations that must be considered when interpreting the findings. First, participants were recruited through non-probability sampling, which limits the generalizability of the results to the broader population. In this context, volunteer bias in sexual research constitutes a systematic source of error that may result in non-representative samples and, consequently, limit the generalizability of findings to the broader population (Dawson et al., 2019). Future studies should aim to employ probabilistic or more diverse sampling strategies to enhance external validity.

Second, the cross-sectional design precludes the establishment of causal relationships between masturbation parameters and sexual arousal in partnered sexual activity. Although the analyses identify significant associations, longitudinal or experimental designs are needed to clarify the directionality of these relationships.

Third, sexual arousal and genital response (i.e., penile erection and vaginal lubrication) were assessed using a single self-report instrument. Although self-report measures are widely used and provide valuable subjective information, they may be influenced by recall bias, social desirability, and limited sensitivity to physiological nuances.

Fourth, the explanatory power of the regression models was relatively modest, particularly in men. This suggests that additional variables not included in the present study—such as relational factors (e.g., relationship satisfaction), psychological variables (e.g., anxiety, body image), or contextual influences—may play a relevant role in sexual arousal and should be considered in future research. Therefore, the observed associations should be interpreted with caution, as they may reflect broader relational, psychological, and sexual profiles rather than the isolated contribution of masturbation parameters.

Fifth, although multiple masturbation parameters were included, the study did not account for qualitative aspects of masturbation practices (e.g., context, motivations, or use of sexual stimuli), which may provide further insight into the relationship between solitary sexual behavior and partnered sexual functioning.

Finally, the sample was restricted to cisgender individuals who reported recent heterosexual activity. Importantly, this inclusion criterion was based on current sexual behavior rather than self-identified sexual orientation. Therefore, the findings cannot be generalized to individuals with diverse patterns of sexual behavior or those with diverse gender identities. Future research should extend this line of inquiry to more diverse populations to examine the applicability of complementary and compensatory models across broader sociocultural and sexual contexts.

## 5. Conclusions

From a clinical perspective, the present findings highlight the importance of conducting an individualized assessment of masturbation parameters when evaluating sexual functioning. The observed variability in how these parameters relate to self-reported sexual arousal in partnered sexual activity of men and women suggests that they do not operate uniformly across individuals, nor do they carry the same explanatory weight. In particular, the consistent association between solitary sexual desire and self-reported sexual arousal underscores the need to carefully consider the conceptual and clinical distinction—and potential overlap—between desire and arousal. Although this overlap has traditionally been emphasized in women, the present findings suggest that it may also be relevant in male sexuality, supporting the need for more flexible and context-sensitive approaches to clinical frameworks.

Overall, this study contributes to a more nuanced understanding of the association between masturbation parameters and self-reported sexual arousal within partnered sexual contexts. The findings revealed gender-differentiated patterns that were differentially consistent with the complementary and compensatory frameworks. In men, the associations between masturbation parameters and partnered sexual arousal were limited and weak, with no significant associations observed for self-reported penile erection. However, solitary sexual desire emerged as a positive correlate of self-reported sexual arousal, which partially nuances this pattern. In contrast, the findings in women were more consistent with the proposed complementary framework, as multiple parameters—including solitary sexual desire, negative attitudes toward masturbation, and the sensory dimension of the subjective orgasm experience—were associated with both general self-reported sexual arousal and self-reported vaginal lubrication.

Taken together, these findings highlight the importance of adopting a multidimensional perspective on sexuality that extends beyond outcome-focused approaches centered solely on increasing arousal. Instead, they emphasize the relevance of subjective, attitudinal, and experiential factors in sexual functioning, particularly in women. Accordingly, future research and clinical practice should consider the complex interplay between these factors to advance understanding and support sexual health. More broadly, these findings reinforce the view that sexual functioning is multifactorial and cannot be explained by a single set of variables.

However, these conclusions should be interpreted with caution given the cross-sectional and correlational design of the study (Wang & Cheng, 2020), which precludes causal inferences regarding whether masturbation enhances sexual functioning within partnered contexts. In addition, these findings apply specifically to cisgender individuals who reported heterosexual sexual activity and prior masturbation experience and, therefore, should not be generalized to other populations.

## Figures and Tables

**Table 1:** Sexual History Characteristics of the participants.

Variable	Total	Men	Women
Age of first masturbation, median (P25–P75)	13 (12–15)	13 (12–14)	14 (12–16)
Negative attitudes toward masturbation, mean (*SD*)	10.82 (2.45)	11.18 (3.03)	10.52 (1.78)
Masturbation frequency, *n* (%)			
Never	20 (1.9%)	5 (1.1%)	15 (2.7%)
Less than once per month	64 (6.2%)	13 (2.8%)	51 (9.1%)
Once or a few times per month	259 (25.2%)	73 (15.6%)	186 (33.3%)
Once or a few times per week	523 (50.9%)	263 (56.1%)	260 (46.5%)
Once per day	116 (11.3%)	85 (18.1%)	31 (5.5%)
More than once a day	46 (4.5%)	30 (6.4%)	16 (2.9%)
Solitary sexual desire, median (P25–P75)	22 (18–26)	23 (19–27)	22 (17–26)
Subjective orgasm experience, mean (*SD*)			
Affective dimension	23.24 (5.34)	22.21 (5.55)	24.13 (4.98)
Sensory dimension	29.67 (16.37)	24.91 (15.41)	33.74 (16.07)
Intimacy dimension	6.28 (3.50)	5.61 (3.34)	6.85 (3.53)
Rewards dimension	11.03 (3.47)	10.87 (3.43)	11.16 (3.51)

*Note*. P25 = 25th percentile; P75 = 75th percentile; *SD* = standard deviation.

**Table 2:** Final Multiple Regression Models Predicting Self-Reported Sexual Arousal and Penile Self-Reported Erection in Men.

Outcome Variable	Predictor	*B*	*SE*	β	95% CI	*t*	*p*	Adj. *R*^2^	VIF
Sexual arousal								.026	
	Solitary sexual desire	0.03	0.01	.20	[0.01, 0.05]	3.52	<.001		1.57
Penile erection								.045	
	Age	−0.02	0.00	−.24	[−0.03, −0.01]	−4.91	<.001		1.15

*Note. B*: unstandardized coefficient; *SE*: standard error; β: standardized coefficient; CI: confidence interval; Adj. *R*^2^: adjusted coefficient of determination; VIF: variance inflation factor. All regression models included age as a covariate, as well as age of first masturbation, negative attitudes toward masturbation, solitary sexual desire, current masturbation frequency, and the dimensions of the subjective orgasm experience as predictors. Only statistically significant predictors are displayed in the table.

**Table 3:** Final Multiple Regression Models Predicting Self-Reported Sexual Arousal and Self-Reported Vaginal Lubrication in Women.

Outcome Variable	Predictors	*B*	*SE*	β	95% CI	*t*	*p*	Adj. *R*^2^	VIF
Sexual arousal								.112	
	Solitary sexual desire	0.04	0.01	.24	[0.02, 0.05]	4.37	<.001		1.90
	Sensory dimension	0.01	0.00	.12	[0.00, 0.01]	2.15	.032		1.83
Vaginal lubrication								.061	
	Negative attitudes	−0.07	0.03	−.09	[−0.13, −0.01]	−2.17	.031		1.05
	Solitary sexual desire	0.03	0.01	.16	[0.00, 0.05]	2.75	.006		1.90
	Sensory dimension	0.01	0.00	.11	[0.00, 0.01]	2.03	.043		1.83

*Note. B*: unstandardized coefficient; *SE*: standard error; β: standardized coefficient; CI: confidence interval; Adj. *R*^2^: adjusted coefficient of determination; VIF: variance inflation factor. All regression models included age as a covariate, as well as age of first masturbation, negative attitudes toward masturbation, solitary sexual desire, current masturbation frequency, and the dimensions of the subjective orgasm experience as predictors. Only statistically significant predictors are displayed in the table.

## Data Availability

The data that support the findings of this study are available from the corresponding author, upon reasonable request.
